# Protein kinetics of superoxide dismutase‐1 in familial and sporadic amyotrophic lateral sclerosis

**DOI:** 10.1002/acn3.51784

**Published:** 2023-04-29

**Authors:** Cindy V. Ly, Margaret D. Ireland, Wade K. Self, James Bollinger, Jennifer Jockel‐Balsarotti, Hillary Herzog, Peggy Allred, Leah Miller, Michael Doyle, Isabel Anez‐Bruzual, Bhavesh Trikamji, Ted Hyman, Tyler Kung, Katherine Nicholson, Robert C. Bucelli, Bruce W. Patterson, Randall J. Bateman, Timothy M. Miller

**Affiliations:** ^1^ Department of Neurology Washington University Saint Louis Missouri USA; ^2^ Sean M. Healey & AMG Center for ALS, Department of Neurology Massachusetts General Hospital Boston Massachusetts USA; ^3^ Department of Medicine Washington University Saint Louis Missouri USA; ^4^ Hope Center for Neurological Disorders Washington University Saint Louis Missouri USA; ^5^ Knight Alzheimer's Disease Research Center Washington University Saint Louis Missouri USA

## Abstract

**Objective:**

Accumulation of misfolded superoxide dismutase‐1 (SOD1) is a pathological hallmark of SOD1‐related amyotrophic lateral sclerosis (ALS) and is observed in sporadic ALS where its role in pathogenesis is controversial. Understanding in vivo protein kinetics may clarify how SOD1 influences neurodegeneration and inform optimal dosing for therapies that lower SOD1 transcripts.

**Methods:**

We employed stable isotope labeling paired with mass spectrometry to evaluate in vivo protein kinetics and concentration of soluble SOD1 in cerebrospinal fluid (CSF) of SOD1 mutation carriers, sporadic ALS participants and controls. A deaminated SOD1 peptide, S**D**GPVKV, that correlates with protein stability was also measured.

**Results:**

In participants with heterozygous SOD1^*A5V*^ mutations, known to cause rapidly progressive ALS, mutant SOD1 protein exhibited ~twofold faster turnover and ~ 16‐fold lower concentration compared to wild‐type SOD1 protein. S**D**GPVKV levels were increased in SOD1^*A5V*^ carriers relative to controls. Thus, SOD1 mutations impact protein kinetics and stability. We applied this approach to sporadic ALS participants and found that SOD1 turnover, concentration, and S**D**GPVKV levels are not significantly different compared to controls.

**Interpretation:**

These results highlight the ability of stable isotope labeling approaches and peptide deamidation to discern the influence of disease mutations on protein kinetics and stability and support implementation of this method to optimize clinical trial design of gene and molecular therapies for neurological disorders.

**Trial Registration:**

Clinicaltrials.gov: NCT03449212.

## Introduction

Amyotrophic lateral sclerosis (ALS) is a neurodegenerative syndrome defined by motor neuron loss in the motor cortex and spinal cord that typically results in death in 3 to 5 years. Although 90% of ALS cases are considered sporadic, about 10% are due to genetic etiology. Nearly 20% of familial ALS (fALS) is caused by toxic gain‐of‐function mutations in Cu/Zn superoxide dismutase‐1 (SOD1). SOD1 consists of 154 amino acids and mutations in over 100 residues have been described to cause ALS though the pathogenicity and penetrance of individual mutations can vary significantly.^1^, ^2^

Misfolded SOD1 aggregates accumulate in motor neurons of patients with SOD1‐related ALS,^3^ and numerous mutations are known to promote SOD1 protein turnover in cell culture and ALS mouse models.^4^, ^5^, ^6^, ^7^ Lower abundance of SOD1 mutant protein in erythrocytes is associated with shorter survival suggesting that protein instability may influence disease progression.^8^ Wild‐type SOD1 is also known to misfold under certain cellular conditions^9^ so a pathogenic role for SOD1 in sporadic ALS (sALS) has been hypothesized. Although some immunohistochemical studies identified SOD1 inclusions in post‐mortem sALS brain and spinal cord,^10^, ^11^ other studies were unable to reproduce these results.^12^, ^13^, ^14^ However, in depth biochemical analysis of post‐mortem familial and sporadic ALS tissue suggests that unstable, mis‐metallated and alternatively post‐translationally modified SOD1 accumulates in ventral spinal cord even in non‐SOD1‐linked cases.^15^ These studies implicate accumulation of misfolded mutant (and possibly wild‐type) SOD1 in ALS pathogenesis. Unraveling factors that modify SOD1 protein kinetics may clarify mechanisms that influence ALS disease pathogenesis.

SOD1‐lowering therapeutics have emerged as a promising strategy for treating patients with SOD1‐associated ALS. Tofersen an antisense oligonucleotide (ASO) targeting SOD1 has completed a Phase III trial and continues to be provided through expanded access.^16^, ^17^ In addition, a strategy involving SOD1 targeting microRNA (miRNA) delivered via adeno‐associated virus (AAV)^18^ was cleared for Phase I/II trial. Both agents target SOD1 mRNA and are predicted to inhibit new synthesis of SOD1.

The ability to measure SOD1 synthesis and clearance in vivo in humans may provide better understanding of the pathogenesis of SOD1 in sporadic ALS and earlier assessments of target engagement in the setting of ASO or other mRNA‐targeted therapeutics.^19^ Stable isotope labeling kinetics (SILK) is a technique that enables the study of protein turnover in vivo.^20^, ^21^ This approach involves labeling participants with a non‐radioactive, stable isotope‐labeled tracer (e.g., [U‐^13^C_6_]L‐leucine) and measuring its incorporation into newly synthesized proteins using quantitative mass spectrometry. SILK has been successfully employed to examine the dynamics of key proteins implicated in Alzheimer's disease including amyloid‐β, tau, and ApoE in human CSF.^22^, ^23^, ^24^, ^25^, ^26^

Using SILK, we previously demonstrated that human CSF SOD1 is a long‐lived protein with a half‐life of ~25 days. In SOD1^*G93A*^ transgenic rats, SOD1 turnover was similar in CSF compared to spinal cord indicating that CSF SOD1 turnover likely reflects SOD1 kinetics in spinal cord.^6^ Furthermore, decreasing SOD1 production in the brain and spinal cord of rodents leads to parallel reduction in CSF SOD1^19^ indicating that CSF SOD1 can be used as a proxy for SOD1 expressed in the central nervous system.

Here, we measure in vivo SOD1 protein kinetics in fALS, sALS, and healthy control participants. We show that mutant SOD1 protein turnover is faster compared to wild‐type protein in SOD1^*A5V*^ mutation carriers, providing in vivo evidence that certain SOD1 mutations can alter protein stability. In addition, SOD1^*A5V*^ protein is expressed at ~6% of the level of wild‐type SOD1. To determine whether altered SOD1 metabolism could play a role in non‐SOD1 ALS, we compared the half‐life of CSF SOD1 protein in sALS and *C9ORF72* hexanucleotide repeat expansion carriers (*C9HRE*) to controls and found no differences suggesting that SOD1 turnover is not altered in non‐SOD1 ALS. These results elucidate the pathological contexts in which altered SOD1 kinetics impacts ALS pathogenesis and paves the way for use of this method to examine the impact of targeted therapeutics on long‐lived proteins.

## Methods

### Cohort recruitment

The study was approved by the Washington University Human Studies Committee (WU IRB# 201207043) and Massachusetts General Brigham Human Research Committee (MGH IRB# 2016P000249). Informed consent was obtained from all participants prior to study inclusion. All research participants had an initial screening visit consisting of a neurological examination and phlebotomy for complete blood count, complete metabolic panel, prothrombin time, and partial thromboplastin time.

Inclusion criteria for controls included age > 18 years and no evidence of ALS by history or examination. ALS participants were eligible if they were > 18 years of age, diagnosed with possible, probable, or definite ALS by El Escorial criteria, and able to tolerate lumbar puncture. Exclusion criteria included dependence on invasive ventilation, contraindication to lumbar puncture (i.e., bleeding disorder, allergy to local anesthetics, evidence of increased intracranial pressure, or skin infection near lumbar puncture site), connective tissue disease, dermatologic disease, adherence to special diet (i.e., gluten‐free), pregnancy, or lab values exceeding twofold upper limit of normal.

Participants with ALS underwent evaluation of ALS functional rating score (ALSFRS‐R) and slow vital capacity at each visit. Blood samples from ALS participants were sent to Prevention Genetics for testing to determine presence of mutations in SOD1 or C9orf72. Participants with sALS were screened and found negative for mutations in C9orf72 and SOD1.

### Stable isotope labeling

Two methods of ^13^C_6_‐leucine labeling were employed in participants, a 10 day oral leucine diet as previously described^6^ followed by a transition to 16 h intravenous ^13^C_6_‐leucine infusion to ease participant burden and improve labelling consistency. Participants in the oral‐labeled leucine cohort received either a controlled leucine diet (2000 mg/ day) prepared by dieticians in the Washington University Research Kitchen or normal diet prepared at home and directed to keep a diet diary for 10 days. The 2000 mg provided in the controlled diet is lower than a typical diet (6000–14,000 mg) but higher than the 1200 mg/ day leucine requirement advised. Labeled U‐[^13^C_6_]L‐leucine (Cambridge Isotope Laboratories, CLM‐2261) was provided as 330 mg of ^13^C_6_‐leucine dissolved in 120 mL Kool‐Aid and given three times a day.

Intravenous ^13^C_6_‐leucine administration was performed using protocols similar to tau SILK studies.^24^ Participants recruited from both study sites were admitted to the Clinical Research Unit (CRU) at Washington University, provided a low‐leucine diet (500–700 mg leucine on day of infusion) and administered ^13^C_6_‐leucine for 16 h at a rate of 4 mg/kg/h. Subjects recruited from MGH returned to MGH for subsequent blood and CSF collections.

### Preparation of ^13^C_6_ ‐leucine‐labeled SOD1 standard curve

^13^C_6_‐labeled SOD1 enrichment standards were prepared by growing HEK293T cells that constitutively express SOD1 in RPMI‐1640 media supplemented with varying ratios of labeled/unlabeled media as previously described.^6^

### Generation of ^15^N‐ and ^14^N‐ recombinant wild‐type and mutant SOD1

A hSOD1 cDNA construct with a N‐terminal GST tag was subcloned into pGEX4T‐1 backbone. Plasmids were propagated in XL‐1 Blue E. coli cells and isolated using Qiagen MIDI prep kits (Qiagen). Site‐directed mutagenesis (QuikChange II, Agilent) was performed to introduce the p.A5V mutation into pGEX4T‐1‐hSOD1. Plasmids were transformed into Rosetta 2 E. Coli (Novagen) to generate recombinant human SOD1 protein.

Bacterial cultures were grown overnight at 37°C in ^15^N‐Celtone complete medium or unlabeled Celtone complete medium (Cambridge Isotope Laboratories), and expression of ^15^N‐ and ^14^N‐ SOD1 recombinant proteins was induced with 1 mM IPTG. Bacterial lysates were incubated with 600 μL glutathione sepharose 4B beads (GE Healthcare) for 30 min. hSOD1 protein was released from GST tag by incubating with 1 unit biotinylated thrombin (Thrombin Cleavage Capture Kit, EMD Millipore) at room temperature for 20 h, followed by addition of 30 μL strepavidin agarose for 30 min to remove thrombin. Supernatant containing hSOD1 protein was collected, quantified by BCA assay, and stored as aliquots at −80°C.

### Analysis of ^13^C_6_ ‐leucine‐labeled SOD1, ^13^C_6_ ‐leucine‐labeled total protein, and plasma‐free ^13^C_6_ ‐leucine by mass spectrometry

Mouse monoclonal anti‐SOD1 antibodies (Sigma‐Aldrich, S2147) were purified and conjugated to M‐270 Epoxy Dynabeads (Invitrogen) at a concentration of 25 μg antibody per 1 mg beads as previously described.^6^ To enable protein quantification, 100 ng of ^15^N SOD1 was added to samples. For analysis of CSF from SOD1 mutation carriers and mutant SOD1 concentration standards, 50 ng of SOD1 and 50 ng of corresponding mutant SOD1 were spiked into samples.

Soluble SOD1 was immunoprecipitated from 1 mL of human CSF collected from participants and HEK293T enrichment standard curve lysates (100 μg protein) using 50 μL of anti‐SOD1 crosslinked Dynabeads. The beads were washed with 25 mM ammonium bicarbonate buffer (AmBic, pH 8.0) (Sigma) twice, and the beads were eluted with 100 μL formic acid (Sigma). Immunoprecipitation, PBS washes, and elution steps were automated and performed in 96‐well format using purification apparatus equipped with magnet head (Kingfisher Flex). The eluent was lyophilized via speedvac (Labconco CentriVap), and isolated SOD1 was resuspended in 25 mM Ambic (pH 8.0) before undergoing reduction with 2.5 mM dithiothreitol (DTT) (Sigma) for 30 min at 37°C followed by alkylation with 7.7 mM iodoacetamide (Sigma) for 30 min at room temperature. LysC (250 ng) was added and allowed to incubate for 4 h followed by incubation with 250 ng Trypsin (Promega) for 16 h at 37°C. Samples were purified using TopTip C18 tip columns and eluted with 66% acetonitrile/ 0.1% formic acid (Fisher Chemical). Samples were lyophilized and resuspended in 50 μL 2.5% acetonitrile/ 1% formic acid prior to liquid chromatography‐mass spectrometry. A TSQ Altis (ThermoFisher) was utilized for all measurements with the exception of SOD1^*A5V*^ analyses which were performed on Lumos Orbitrap (ThermoFisher).

Tracer‐to‐tracee ratios (TTR) were obtained by comparing the area under the curve of ^13^C_6_‐leucine and ^12^C_6_‐leucine signal for leucine‐containing SOD1 peptides derived from LysC/ Trypsin digestion and converted to mole fraction labeled (MFL) using the equation, MFL = TTR/ (1 + TTR). One to three transition ions for each peptide were selected for acquisition based on strong signal correlation.

Plasma‐free ^13^C_6_‐leucine TTR (which represents the precursor pool enrichment for SOD1 protein synthesis) was measured as tert‐butyldimethylsilyl (tBDMS) derivatives by electron impact ionization gas chromatography–mass spectrometry (GC/ MS) as previously described.^24^ CSF total protein ^13^C_6_‐leucine isotopic enrichment was measured as previously described.^6^

### Compartmental modeling of protein kinetics

A compartmental model was used to assess the kinetics of CSF SOD1 and total protein. A forcing function of the plasma leucine ^13^C_6_‐leucine time course (a linear interpolation of sampled time points from *t* = 0 through 18 h connected to a sum of 3 exponentials function that defined plasma leucine kinetics from 18 h to the last CSF time point) was used as an input into a system of two serial compartments with identical fractional turnover rates to represent either CSF SOD1 or total protein. Some direct input of newly synthesized protein into the second of the two compartments was required to optimally fit the protein enrichment time courses. Parallel arms of the model implicitly tracked the change in labeled and unlabeled proteins from an initial steady state consisting of only unlabeled protein as the MFL of plasma leucine changed over time, and an adjustable scaling factor was used to account for in vivo isotopic dilution and/or residual errors in the accuracy of SOD1 peptide enrichment measurements. The whole system residence time (days) and fractional turnover rate (FTR, pools/day) were determined using the SAAM modeling program (The Epsilon Group, Charlottesville, VA). Production rates (ng/mL/day) were calculated as the product of FTR and concentration. Half‐life was calculated as ln[2]/ FTR.

### SDGPVKV peptide analysis

The SDGPVKV peptide was measured from 50 μL aliquots of CSF by Clarus Analytical as previously described.^27^

### Statistics

Statistical analyses were performed using GraphPad Prism 7.0 (GraphPad). Quantitative data are represented as mean ± SD. Intergroup comparisons of mean values were conducted using two‐tailed t‐tests assuming normal distribution of the data. Statistical analysis of S**D**GPVKV levels was conducted using one‐way ANOVA. P‐values <0.05 were considered significant. Spearman correlation analyses were used to determine correlation between SOD1 kinetic and clinical parameters.

## Results

### Clinical cohort

We examined six controls, four sALS, and one participant with a SOD1 mutation (p. A5V) who underwent 10 day oral administration of ^13^C_6_‐leucine (Table 1). We also infused ^13^C_6_‐leucine intravenously over 16 h in a parallel cohort comprised of five controls, 11 sALS, two *C9HRE* carriers, and three SOD1‐mutation carriers (p. A5V) (Table 2). Controls recruited into the study were healthy and did not have neurological illness by history or examination. Three of the four SOD1^*A5V*^ carriers were asymptomatic. Detailed kinetic analysis was not available from one SOD1^*A5V*^ ALS participant (ALS13) who was only able to complete lumbar punctures at days 8, 15, and 29 post‐^13^C_6_‐leucine infusion before withdrawal from the study.

**Table 1:** Clinical characteristics of cohort receiving orally administered ^13^C_6_‐leucine.

	Participant	Cohort	Age	Gender (%male)	BMI	Site of onset (% limb)	Disease duration (months)	Initial ALSFRS	ALSFRS decline (/month)	Initial SVC (L)	SVC decline (%/month)
1	NC02	control	50	M	19.5						
2	NC03	control	66	M	25.4						
3	NC05	control	32	M	22.7						
4	NC06	control	72	M	30.2						
5	NC07	control	74	M	27.3						
6	NC08	control	60	F	30.1						
7	NC54	control	48	F	30.8						
8	NC56	control	60	M	24.3						
	Mean ± SD		58 ± 14	75%	26.3 ± 4.1						
9	ALS01	sALS	65	M	25.6	Limb	55	36	−0.83	3.8	−7.6
10	ALS52	sALS	64	M	25.1	Bulbar	59	35	−2.11	4.2	−4.0
11	ALS53	sALS	66	M	27.2	Limb	20	42	−0.02	3.2	7.2
12	ALS56	sALS	62	M	26.1	Limb	69	28	−0.27	3.4	−4.1
13	Mean ± SD		64 ± 1.7	100%	26 ± 0.9	75%	50.8 ± 21.3	35.3 ± 5.7	−0.81 ± 0.93	3.6 ± 0.5	−2.5 ± 6.1
14	ALS51	SOD1^*A5V*^	56	M	37.5	n/a	n/a	48	0	n/a	n/a

ALSFRS‐R, revised ALS functional rating score; Asym, asymptomatic; BMI, body mass index; F, female; L, liter; M, male; n/a, not available; sALS, sporadic amyotrophic lateral sclerosis; SD, standard deviation; SOD1, superoxide dismutase‐1; SVC, slow vital capacity.

**Table 2:** Clinical characteristic of cohort receiving 16 h intravenous ^13^C_6_‐leucine.

	Participant	Cohort	Age	Gender (%male)	BMI	Site of onset (% limb)	Disease duration (months)	Initial ALSFRS	ALSFRS decline (/month)	Initial SVC (L)	SVC decline (%/month)
15	NC10	control	60	F	35.7						
16	NC13	control	43	F	32.5						
17	NC14	control	67	M	27.8						
18	NC15	control	61	F	22.0						
19	NC16	control	68	M	30.1						
	Mean ± SD		60 ± 10	40%	29.6 ± 5.2						
20	ALS04	sALS	64	M	19.4	Limb	25	28	−0.61	3.2	−9.1
21	ALS06	sALS	62	M	29.3	Limb	8	42	−0.58	4.0	−2.5
22	ALS07	sALS	56	M	34.2	Bulbar	8	43	−2.54	1.8	10.0
23	ALS10	sALS	65	F	24.4	Limb	35	27	−0.57	1.8	−5.5
24	ALS11	sALS	63	F	21.2	Limb	68	35	−0.20	2.2	−4.5
25	ALS12	sALS	69	M	26.5	n/a	62	33	0.21	2.9	3.4
26	ALS14	sALS	58	M	26.9	Limb	16	42	−0.56	3.7	2.1
27	ALS15	sALS	57	F	26.1	n/a	n/a	33	n/a	2.0	3.5
28	ALS17	sALS	64	M	33	Limb	69	36	−0.19	3.9	1.5
29	ALS57	sALS	58	F	21.3	Limb	39	44	−0.09	3.4	0.59
30	ALS60	sALS	50	M	28.3	Limb	46	40	−0.24	4.8	−3.1
	Mean ± SD		61 ± 5.3	64%	26.4 ± 4.7	73%	37.5 ± 23.5	36 ± 6	−0.58 ± 0.72	3.1 ± 1.0	−0.7 ± 4.5
31	ALS05	C9‐ALS	69	F	21.4	Bulbar	37.4	37	−0.27	2.2	−10.5
32	ALS09	C9‐FTD	63	M	33.1	n/a	n/a	48	0	3.7	0.73
	Mean ± SD		66 ± 4.2	50%	27.5 ± 8.3	0%		43 ± 8	−0.14 ± 0.19	3.0 ± 1.1	−3.3 ± 6.0
33	ALS13	SOD1^*A5V*^	40	M	26.3	Limb	8	45	0	5.9	−6.8
34	ALS20	SOD1^*A5V*^	41	F	22.3	n/a	n/a	n/a	0	n/a	n/a
35	ALS25	SOD1^*A5V*^	63	M	25.1	n/a	n/a	n/a	0	n/a	n/a
	Mean ± SD		48 ± 13	67%	24.6 ± 2.1	33%			0		

ALSFRS‐R, revised ALS functional rating score; Asym, asymptomatic; BMI, body mass index; C9, C9orf72; F, female; FTD, frontotemporal dementia; L, liter; M, male; n/a, not available; sALS, sporadic amyotrophic lateral sclerosis; SD, standard deviation; SOD1, superoxide dismutase‐1; SVC, slow vital capacity.

Clinical characteristics including disease duration, initial ALSFRS‐R, ALSFRS‐R decline, site of onset, SVC decline, and BMI were recorded for participants with sALS. Controls (*n* = 8) and sALS (*n* = 4) participants in the oral cohort were age‐matched (control: 58 ± 14 years, sALS: 64 ± 1.7, *p* = 0.329) with a larger male predominance in the sALS cohort (Table 1). Similarly, controls (*n* = 5) and sALS participants (*n* = 11) in the infused cohort were age‐matched (control: 60 ± 10 years, sALS: 61 ± 5.3 years, *p* = 0.887) though the sALS cohort was more male‐predominant (Table 2).

### Modification of SILK method to measure turnover of SOD1 mutant peptides

**Figure 1:**
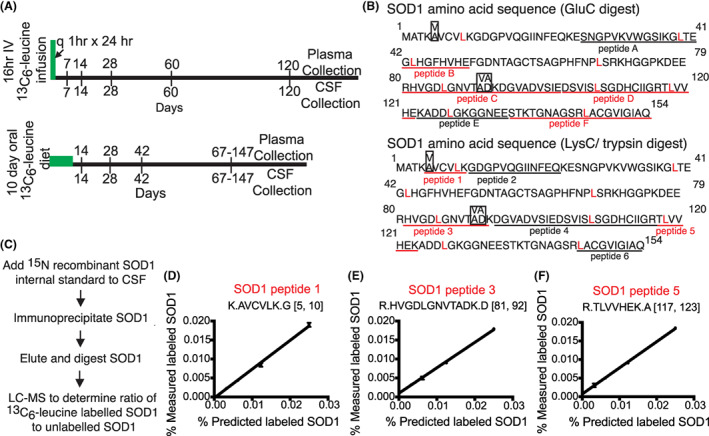
Isolation and measurement of heavy‐labeled human SOD1 in CSF by mass spectrometry. (A) Schematic of 16‐h intravenous ^13^C_6_‐leucine infusion and 10‐day oral ^13^C_6_‐leucine diet (bottom) labeling paradigms. CSF and plasma were collected at the designated time points. (B) Amino acid sequence of SOD1 protein depicting digested peptides using GluC or LysC/ trypsin digestion strategies. Quantifiable peptides (underline) and measurable leucine‐containing heavy‐labeled peptides (red underline) are shown. SOD1 mutations represented within our cohort are indicated (boxes). (C) Flow chart detailing steps taken to process CSF including addition of recombinant ^15^N‐SOD1 internal standard, immunoprecipitation, elution and digestion, and LC–MS/MS analysis. LC–MS/MS standard curves for leucine‐containing peptides used to quantify labeled SOD1, (D) peptide 1: AVCVLK (E) peptide 3: HVGDLGNVTADK (F) peptide 5: TLVVHEK. Bracketed numbers denote amino acid boundaries for peptides. CSF—cerebrospinal fluid, LC–MS/MS—liquid chromatography–tandem mass spectrometry.

Our prior technique employed GluC digestion of captured SOD1 protein which generates four reliably measurable leucine‐containing peptides (peptide B: ^42^GLHGFHVHE^50^, peptide C: ^80^RHVGDLGNVTADKDGVADVSIE^101^, peptide D: ^102^DSVISLSGDHCIIGRTLVVE^122^, peptide F: ^135^STKTGNAGSRLACGVIGIAQ^154^) and six peptides used for protein quantification (Fig. 1A–C).^6^ However, this method was unable to isolate N‐terminal peptides that express the SOD1^*A5V*^ mutation, the most common SOD1 mutation found in ~30% of SOD1‐ALS patients.^2^ Thus, we developed a digestion strategy using LysC/ trypsin that liberated an N‐terminal leucine‐containing peptide harboring the SOD1^*A5V*^ mutation represented within our cohort, peptide 1: ^5^AVCVLK^10^. Six peptides could potentially be used to quantify SOD1 protein using this method (Fig. 1B). We identified three leucine‐containing peptides that accurately approximated tracer incorporation using ^13^C_6_‐leucine‐labeled HEK293T cells (peptide 1, peptide 3: ^81^HVGDLGNVTADK^92^, and peptide 5: ^117^TLVVHEK^123^) (Fig. 1D–F). Combining the two digestion methods, nearly 52% of SOD1 residues are embedded within measurable leucine‐containing peptides and amenable to analysis of mutant peptide turnover.

### SOD1^A5V^ has a shorter half‐life compared to SOD1^WT^

**Figure 2:**
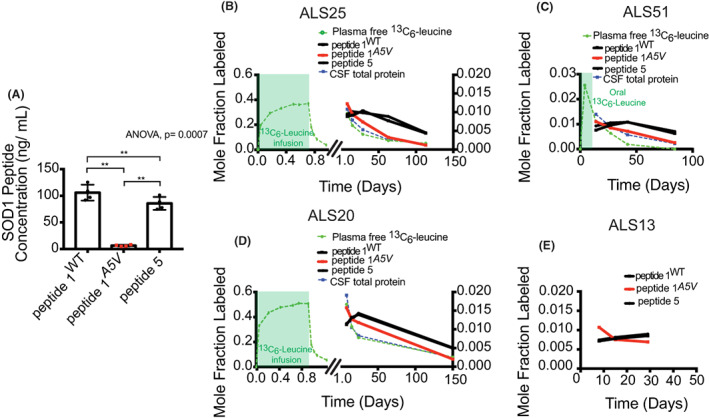
Mutant SOD1^*A5V*^ has a shorter half‐life than SOD1^WT^ in human CSF. (A) CSF concentration of peptide 1^*A5V*^ is markedly reduced compared to peptide 1 and peptide 5. Statistics by one‐way ANOVA, *p* = 0.0007 followed by Tukey's multiple comparison's test, ** denotes 0.01 < *p* < 0.001. Mole fraction labeled plasma‐free ^13^C_6_‐leucine, CSF total protein, peptide 1, peptide 1^*A5V*^, and peptide 5 plotted over time in (B) ALS25 (C) ALS51, and (D) ALS20 participants. (E) Mole fraction labeled peptide WT1, peptide 1^*A5V*^, and peptide 5 with shortened time course in ALS13 participant shows divergence of mutant and WT peptide kinetics.

To examine turnover of mutant SOD1 protein, we assessed SOD1 kinetics in participants with SOD1^*A5V*^ mutations. The concentration of peptide 1^*A5V*^ and peptide 1^WT^ proteins was also measured to determine the stability of mutant protein. The concentration of peptide 1^*A5V*^ in CSF was ~16‐fold lower than its wild‐type counterpart ([peptide 1^WT^] = 105.9 ± 14.9 ng/ mL; [peptide 1^*A5V*^] = 6.4 ± 1.4 ng/ mL; *n* = 4) and ~ 13‐fold lower than peptide 5 ([peptide 5] = 85.7 ± 12.1 ng/ mL, *n* = 4, ANOVA *p* = 0.0007) (Fig. 2A). The amount of peptide 5, which represents a pool of wild‐type and mutant SOD1 protein, was similar to peptide 1^WT^, reflecting the small contribution of SOD1^*A5V*^ to total CSF SOD1 concentration.

Reduced levels of mutant protein can occur due to increased clearance via increased protein degradation, accumulation in aggregates, decreased production, or reduced cellular release into CSF. The fractional turnover rate is increased by ~twofold for peptide 1^*A5V*^ relative to peptide 1^WT^ and peptide 5 suggesting that mutant protein turns over more rapidly (Table 3). In participants harboring SOD1^*A5V*^, peptide 1^*A5V*^ half‐life was shorter compared to native peptide 1^WT^, showing that SOD1^*A5V*^ protein is turned over ~twofold faster than SOD1^WT^ (Table 3, Fig. 2B–E). The turnover of peptide 1^WT^ was similar to peptide 5, a C‐terminal SOD1 peptide, in SOD1^*A5V*^ participants as well as control participants (Table 3). Participant ALS13 exhibited divergence in SOD1^*A5V*^ and SOD1^WT^ peptide behavior similar to other SOD1^*A5V*^ participants but did not undergo kinetic modeling due to an abbreviated time course (Fig. 2E). Furthermore, the production rate of peptide 1^*A5V*^ is ~15–18% that of peptide 1^WT^ and peptide 5 indicating that SOD1^*A5V*^ protein has a lower rate of production and/or cellular release compared to SOD1^WT^ (Table 3). Thus, the ~16‐fold reduced levels of mutant protein are due to ~twofold faster turnover of SOD1^*A5V*^ as well as significantly reduced production or release into CSF.

**Table 3:** SOD1 kinetic parameters in sporadic and familial ALS.

	FTR (pools/day)	SOD1 Half‐life (days)	Production Rate (ng/mL/day)	Total Protein Half‐life (days)
16 h IV ^13^C_6_‐leucine infusion				
Control (*n* = 5)	0.0211 ± 0.0019 (0.0187–0.0231)	33.1 ± 3.0 (30.1–37.0)	1.30 ± 0.38 (0.64–1.60)	13.6 ± 1.8 (11.4–15.7)
sALS (*n* = 11)	0.0355 ± 0.0055 (0.0189–0.0275)	32.6 ± 3.7 (25.2–36.6)	1.46 ± 0.88 (0.40–3.59)	12.2 ± 2.6 (8.2–15.7)
C9 (*n* = 2)	0.0334 ± 0.0003 (0.0204–0.0211)	33.5 ± 0.8 (32.9–34.1)	0.887 ± 0.033 (0.863–0.910)	11.1 ± 0.63 (10.6–11.5)
10‐day oral ^13^C_6_‐leucine diet				
Control (*n* = 7)	0.0295 ± 0.0069 (0.0214–0.0399)	24.6 ± 5.8 (17.4–32.4)	2.06 ± 0.29 (1.45–2.34)	6.0 ± 3.7 (2.9–13.1)
sALS (*n* = 4)	0.0251 ± 0.0056 (0.0170–0.0295)	28.9 ± 8.0 (23.5–40.7)	1.36 ± 0.70 (0.90–2.4)	6.9 ± 4.0 (4.0–12.9)
SOD1^A5V^ (16‐h IV ^13^C_6_‐leucine infusion) (*n* = 2)				
Peptide 1^WT^	0.024 ± 0.001 (0.023–0.025)	29.0 ± 1.4 (28.0–30.0)	2.59 ± 0.44 (2.27–2.90)	
Peptide 1^*A5V*^	0.056 ± 0.003 (0.054–0.058)	12.4 ± 0.6 (12.0–12.9)	0.38 ± 0.12 (0.30–0.47)	
Peptide 5	0.023 ± 0.001 (0.022–0.024)	30.1 ± 1.9 (28.8–31.4)	2.02 ± 0.31 (1.80–2.24)	
CSF total protein	0.093 ± 0.042 (0.064–0.123)		23,970.30 ± 3,454.78 (21,527.40 – 26,413.20)	8.2 ± 3.7 (5.6–10.9)
SOD1^A5V^ (10‐day IV ^13^C_6_‐leucine infusion) (*n* = 1)				
Peptide 1^WT^	0.018	38.2	1.98	
Peptide 1^*A5V*^	0.029	23.9	0.20	
Peptide 5	0.015	44.9	1.37	
CSF Total Protein	0.042		30,804.39	16.6

Fractional turnover rate (FTR), CSF SOD1 half‐life, CSF SOD1 production rate (ng/mL/day), and CSF total protein half‐life for 16‐h ^13^C_6_‐leucine infusion and 10‐day oral ^13^C_6_‐leucine in control, sALS, and SOD1^*A5V*^‐ALS cohorts. For SOD1‐ALS kinetics, model‐derived half‐life is provided for wild‐type and corresponding mutant SOD1 peptides. Values are provided as mean ± standard deviation. Range of values provided in parentheses.

### SOD1 kinetics are not altered in sALS cohort

**Figure 3:**
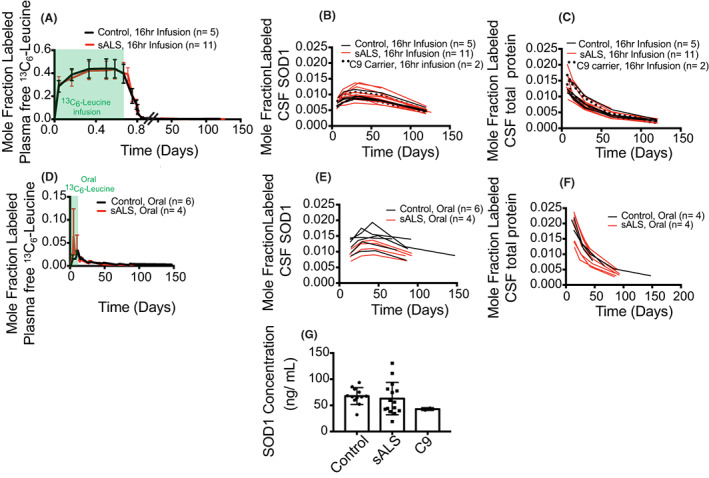
SOD1 kinetics is not altered in non‐SOD1 ALS. Healthy control, sporadic ALS (sALS), and C9orf72 mutation carriers (C9+) were labeled with ^13^C_6_‐leucine. Mole fraction labeled (A) plasma‐free ^13^C_6_‐leucine and (B) CSF SOD1 and (C) CSF total protein plotted over time for control (black line), sALS (red line), and C9+ (black dotted line) participants who were labeled using 16‐h intravenous infusion. Mole fraction labeled (D) plasma‐free ^13^C_6_‐leucine (E) CSF SOD1 and (F) CSF total protein plotted over time for control (black line) and sALS (red line) participants who were labeled using 10‐day oral labeling. Mole fraction labeled SOD1 was derived from the mean of three peptides (peptide 1, peptide 3, and peptide 5). Green highlighted regions denote period of ^13^C_6_‐leucine labeling. (G) CSF SOD1 concentration in control, sALS, and C9+ participants. Error bars denote standard deviation.

To determine whether altered SOD1 metabolism plays a role in non‐SOD1 ALS, we compared the kinetics of CSF SOD1 protein in ALS to controls. Plasma enrichment of ^13^C_6_‐leucine was similar between control and sALS cohorts for either infused or oral tracer (Fig. 3A,D) and reflect the available labeling pool. At peak, ~1–2% of SOD1 protein (Fig. 3B,E) and CSF total protein (Fig. 3C,F) was isotopically labeled in control, sALS, and *C9HRE* participants using both labeling protocols. The concentration of SOD1 was quantified and found to be similar between sALS and controls (sALS [SOD1] = 63.0 ± 30.9 ng/mL, *n* = 15; control [SOD1] = 67.7 ± 16.0 ng/mL, *n* = 12, *p* = 0.639) (Fig. 3G).

Within the ^13^C_6_‐leucine infused cohort, SOD1 half‐life was similar in sALS, *C9HRE* participants, and controls (Table 3). SOD1 half‐life in oral‐labeled sALS and control participants was also not significantly different (Table 3). The half‐life of the total CSF protein pool was twofold to fourfold faster compared to SOD1 consistent with prior studies,^6^ indicating that CSF SOD1 is a relatively long‐lived protein (Table 3). Together, these data indicate that the kinetics and concentration of soluble CSF SOD1 are not altered in sALS.

### Correlations between sALS clinical characteristics and SOD1 half‐life

Correlations between SOD1 kinetic parameters and sALS clinical characteristics including age, body mass index (BMI), rate of ALSFRS‐R decline, and disease duration were examined for the infused ^13^C_6_‐leucine cohort. SOD1 kinetic parameters examined were SOD1 half‐life, total protein half‐life, SOD1 production rate, and SOD1 concentration. SOD1 half‐life correlated with SVC decline (Spearman's *r* = 0.736, *p* = 0.013) but no other clinical characteristics. In addition, ALSFRS‐R decline correlated with SOD1 concentration (Spearman's *r* = 0.673, *p* = 0.039). However, correlations of ALSFRS‐R decline with SOD1 production rate (Spearman's *r* = 0.588, *p* = 0.081) and total protein half‐life (Spearman's *r* = −0.576, *p* = 0.088) did not reach significance. Notably, the rate of ALSFRS‐R decline in this cohort was relatively slow (mean ALSFRS‐R decline = 0.60 ± 0.72 points per month) and only included one fast progressor (ALSFRS‐R decline = 2.54 per month), limiting interpretation of these findings.

### Deamidated SOD1 peptide, S**D**GPVKV, is elevated in SOD1 mutation carriers but not in controls or sALS

**Figure 4:**
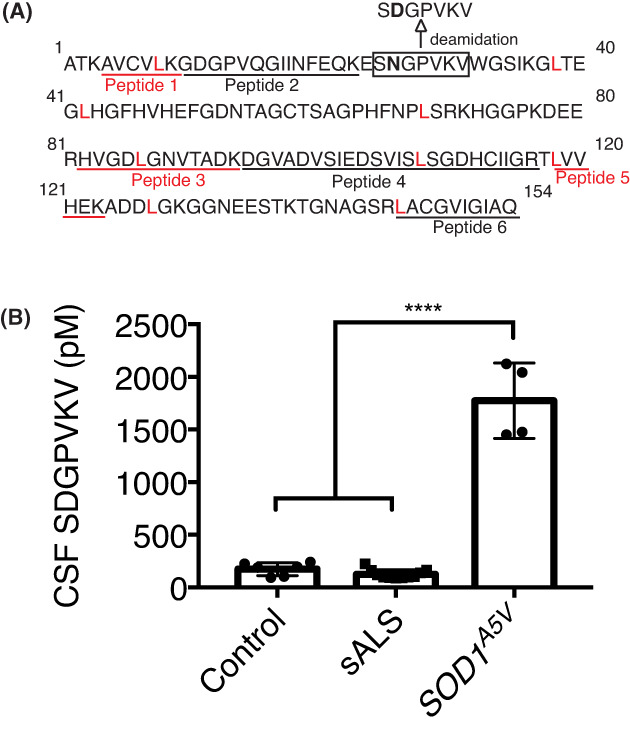
Deamidated SOD1 peptide, SDGPVKV, is elevated in SOD1^*A5V*^ carriers but not in controls or sALS. (A) Amino acid sequence of SOD1 protein depicting location of deaminated peptide in relation to quantified and measurable ^13^C_6_‐leucine labeled SOD1 peptides. (B) Quantitation of CSF S**D**GPVKV peptide in control, sALS, and SOD1^*A5V*^ carriers. ANOVA *p* < 0.0001.

An endogenous SOD1 peptide, S**D**GPVKV, was previously identified as a product of intracellular SOD1 degradation whose levels correlated with in vivo protein stability and degradation.^27^ S**D**GPVKV is a deaminated product of the S**N**GPVKV sequence (amino acids 25–31) of SOD1 (Fig. 4A). Using in silico tools, the sequence was predicted to derive from 20S proteasomal degradation and shown to undergo post‐proteolytic asparagine deamidation at N^26^. Asparagine deamidation is promoted by the oxidative environment of neurodegenerative conditions and associated with protein misfolding.

S**D**GPVKV levels are increased in spinal cords of SOD1^*G93A*^ mice and were found to be elevated in the CSF of SOD1 mutation carriers compared to controls in a manner that correlates to SOD1 stability changes conferred by individual mutations.

To determine whether changes in SOD1 half‐life are associated with reduced SOD1 stability, S**D**GPVKV peptide was measured in CSF of control, sALS, and SOD1^*A5V*^ carriers. S**D**GPVKV levels in SOD1^*A5V*^ carriers were significantly increased (1,774.0 ± 359.1, *n* = 4) compared to sALS (124.9 ± 42.7, *n* = 11), and controls (173.7 ± 61.5, *n* = 6) (ANOVA, *p* < 0.0001).

## Conclusions

Protein aggregation and impaired proteostasis are pathogenic mechanisms that underlie numerous neurodegenerative disorders. SILK is a method that offers a window to examine in vivo turnover (synthesis and clearance) of key proteins and how these processes are affected in neurodegenerative diseases characterized by misfolding and accumulation of pathogenic proteins. Moreover, measures of protein kinetics can inform optimal dosing schedules for therapies in clinical trials and provide improved biomarkers for assessing target engagement.

We utilized SILK methods to measure in vivo turnover of CSF SOD1 protein in SOD1^*A5V*^ mutation carriers. SOD1^*A5V*^ mutations comprise ~50% of SOD1‐ALS cases in North America and cause a form of the disease with rapid progression and average survival of 1.4 years. We demonstrate that mutant protein turnover is rapid compared to corresponding wild‐type protein, demonstrating for the first time a divergence in kinetics between mutant and wild‐type protein alleles in an inherited neurodegenerative disease. This finding corroborates in vitro and SOD1 rodent model studies that demonstrate faster turnover of mutant protein.^4^, ^6^, ^7^ Three of the SOD1^*A5V*^ participants were asymptomatic, so SOD1 kinetic changes are evident prior to clinical onset. This observation mirrors findings from SOD1^*G85R*^ mutant mice showing that SOD1^*G85R*^ protein turns over faster than SOD1^WT^ even prior to development of clinical symptoms.^7^ Aggregates in SOD1^*G85R*^ mice have also been shown to develop before onset of clinical symptoms and are among the first pathological signs of disease.^28^ Thus, altered SOD1 kinetics may signify propensity to develop disease suggesting that this approach may be able to identify variants of unknown significance in SOD1 that are likely pathogenic.

Although the turnover of SOD1^*A5V*^ is ~twofold faster, the concentration of CSF SOD1^*A5V*^ protein is ~16‐fold lower than SOD1^WT^. Faster turnover does not solely account for observed differences in mutant and wild‐type protein providing support that mutant protein is not only unstable but undergoes reduced synthesis and/or cellular release into CSF. Prior studies that examined erythrocytes from SOD1 mutation carriers similarly found mutant protein was reduced or undetectable for a subset of variants, including SOD1^*A5V*^ .^8^ Erythrocytes are enucleated cells devoid of protein synthesis, a property likely to highlight instability of mutant SOD1. The accelerated turnover and reduced stability of SOD1^*A5V*^ protein is consistent with a model in which mutated protein misfolds and is cleared via proteostatic mechanisms or incorporated within aggregates.

To further assess the pathogenicity of SOD1^*A5V*^ and examine stability of SOD1 in sALS, we measured an endogenous deamidated SOD1 peptide, S**D**GPVKV, previously shown to reflect the degree of misfolding conferred by distinct mutations.^27^ S**D**GPVKV levels were markedly elevated in symptomatic and asymptomatic SOD1^*A5V*^ suggesting a link between the high propensity of the protein to misfold and aggressive phenotype of this mutation. Further clinico‐pathologic studies correlating CSF S**D**GPVKV to SOD1 aggregate burden and disease progression in SOD1 mutation carriers are warranted to define the nature of this biomarker.

Nearly 150 mutations in SOD1 have been described. Pathogenic SOD1 mutations are hypothesized to cause destabilization, misfolding, and aberrant toxic aggregation of mutant and wild‐type protein. Multiple in vitro and in vivo studies have characterized the chemically and structurally diverse consequences of SOD1 mutations.^29^ Although factors including aggregation potential, protein stability, and potential for mutant‐to‐wild‐type heterodimerization have been associated with disease progression,^8^, ^30^, ^31^, ^32^ they are not altogether predictive of clinical phenotype. Given the variety of variants in SOD1, approaches are needed to ascertain disease‐causing mutations to facilitate identification of SOD1 mutation carriers for clinical trials of SOD1‐lowering therapies. Expanding the array of SOD1 mutations examined on the basis of kinetic behavior will further clarify determinants of pathogenicity. Correlation of kinetic parameters to SOD1 inclusion burden will also be beneficial. However, determining inclusion burden is challenging as PET‐based modalities that employ aggregate‐binding radiotracers have not been developed for SOD1 aggregates and post‐mortem analysis may be temporally dissociated from relevant measures. Nonetheless, the methods developed to measure the concentration as well as turnover of mutant and wild‐type SOD1 provide a versatile platform for characterizing pathogenicity of SOD1 variants in vivo.

The involvement of SOD1 aggregates in sALS is controversial as some studies have identified SOD1 inclusions in brain and spinal cord from sALS patients by immunohistochemistry.^10^, ^11^ However, other studies were unable to recapitulate these findings.^12^, ^13^, ^14^ Recent biochemical and proteomic analysis of post‐mortem tissue suggests that disordered SOD1 conformers associated with mis‐metallation, altered post‐translational modifications, and loss of enzymatic activity accumulate in the ventral spinal cord of SOD1‐linked and non‐SOD1 linked ALS.^15^ Misfolding of native SOD1 in sALS would suggest a common disease mechanism between familial and sporadic forms of the disease that could be targeted by SOD1‐lowering therapies. Our results indicate that wild‐type SOD1 turnover is not altered in the CSF of non‐SOD1 ALS compared to controls. We also determined that CSF SOD1 levels are similar between sALS and controls consistent with studies that employed immunoassay‐based methods.^33^, ^34^ Notably, the levels of deaminated SOD1 peptide in sALS did not differ from controls suggesting that the stability of SOD1 may not be principally affected in sporadic cases. Together, these findings indicate that turnover of soluble SOD1 is not significantly altered in non‐SOD1‐ALS. Nearly 80% of CSF proteins derive from blood while only ~20% are neuronally derived.^35^ Although the proportion of blood‐derived SOD1 in CSF is unknown, its presence may obscure the contribution of disordered SOD1 from spinal cord. Given this caveat, we are unable to exclude a contribution of native SOD1 to sALS pathogenesis based on these studies.

Several limitations complicate interpretation of these results. First, the sample size of SOD1 mutation carriers, non‐SOD1 ALS, and control cohorts was relatively small. Second, we observed more rapid turnover of the CSF total protein pool in orally labeled compared to infused cohorts so these groups were analyzed separately. This suggests that ^13^C_6_‐leucine labeling paradigms may label distinct subsets of proteins with different kinetic profiles, or that the compartmental model that uses plasma leucine enrichment is unable to resolve local (in situ) tracer recycling issues that affect the accuracy of assessing protein FTR. Third, the SILK method involves immunoprecipitation of SOD1 using antibodies to native SOD1 and may not fully capture modified SOD1 species such as misfolded SOD1. However, we were able to quantitatively measure mutant SOD1 protein and discern rapid turnover of SOD1^*A5V*^. In addition, employing antibodies to native SOD1 allows application of the assay to pharmacodynamic studies of SOD1‐lowering therapies that target wild‐type SOD1. Assay modifications that allow specific targeting of misfolded SOD1 or PTMs associated with disordered SOD1 conformers may improve sensitivity for detecting altered protein turnover. Fourth, the factors that dictate release of SOD1 into CSF are not well understood, and further studies are needed to elucidate these mechanisms including the role of passive (i.e., neurodegeneration) and/or active processes (i.e., secretory release) in release, contribution of glymphatic clearance, and cellular source of CSF SOD1.

SOD1‐lowering strategies that reduce SOD1 mRNA and inhibit synthesis of new SOD1 protein have emerged as promising therapeutic approaches for SOD1‐ALS. In the Phase III trial of tofersen in SOD1‐ALS, CSF SOD1 protein declined by ~30% at 12 weeks in the high‐dose group.^16^ Given the long half‐life of SOD1 of ~30 days, reductions in total CSF SOD1 protein would be expected to lag ASO‐mediated inhibition of SOD1 mRNA and reduced protein synthesis. Notably, SOD1^*G93A*^ rats treated with SOD1‐lowering ASO have decreased rates of wild‐type SOD1 protein synthesis that were observed prior to decreases in total CSF SOD1 protein.^19^ The ability to characterize kinetic behavior of mutant SOD1 protein advances use of this method to clarify impact of ASO treatment on mutant protein concentration, the primary toxic therapeutic target, and improve clinical trial design. We propose that applying protein kinetic analysis to clinical trials has the potential to establish earlier pharmacodynamic biomarkers of target engagement and guide optimal timing and dosing for mRNA‐directed therapeutics.

## Author Contributions

CVL designed and conducted SOD1 immunoprecipitation and plasma leucine GC/MS experiments, acquired clinical data, analyzed data, and wrote the manuscript. MDI assisted with SOD1 immunoprecipitation experiments, processed human biofluid samples, conducted and analyzed plasma leucine GC–MS data, and edited the manuscript. WS helped develop SOD1 SILK methods, contributed intellectual guidance, and edited the manuscript, JB provided intellectual guidance and acquired mass spectrometric data. JJB, HH, PA, LM, MD, and IAB provided research coordination and acquired participant demographic data, BT acquired clinical data. TK ascertained quality control of clinical specimens. KN provided site coordination of the clinical study at MGH. RB acquired clinical data. BWP and RJB provided intellectual guidance and edited the manuscript and RJB supervised the SOD1 measures including method development and edited the manuscript. TMM supervised the study, provided intellectual guidance, provided financial support, and edited the manuscript.

## Funding information

Funding was provided by NIH K08 NS107621 (C.V. Ly), NIH R01 NS097816 (T.M. Miller), the Tracy Family SILQ Center (R.J. Bateman), and NIH P30 DK056341 (Washington University Nutrition and Obesity Research Center; B.W. Patterson).

## Conflict of Interest

CVL has received research funding from Target ALS and consulting fees from Biogen. KN has received research funding from AI Therapeutics and Alector Therapeutics, and consulting fees from Biogen, MT Pharma of America, Regeneron, and Amylyx Therapeutics while employed by Massachusetts General Hospital. KN is now employed by Sanofi. RCB receives consulting fees from Biogen and receives research funding from Biogen and Ionis Pharmaceuticals for clinical trials. RCB serves on an advisory board for Biogen and has equity in Neuroquestions. RJB has received research funding from Avid Radiopharmaceuticals, Janssen, Roche/Genentech, Eli Lilly, Eisai, Biogen, AbbVie, Bristol Myers Squibb, and Novartis. Washington University and RJB have equity ownership interest in C2N Diagnostics and receive income based on technology (stable isotope labeling kinetics, blood plasma assay, and methods of diagnosing AD with phosphorylation changes) licensed by Washington University to C2N Diagnostics. RJB receives income from C2N Diagnostics for serving on the scientific advisory board. TMM has licensing agreements with C2N Diagnostics and Ionis Pharmaceuticals and receives consulting fees from Biogen, Biolo, LLC, Cytokinetics, Disarm, Ionis Pharmaceuticals, and UCB.
